# A Novel Peroxidase *CanPOD* Gene of Pepper Is Involved in Defense Responses to *Phytophtora capsici* Infection as well as Abiotic Stress Tolerance

**DOI:** 10.3390/ijms14023158

**Published:** 2013-02-04

**Authors:** Jun-E Wang, Ke-Ke Liu, Da-Wei Li, Ying-Li Zhang, Qian Zhao, Yu-Mei He, Zhen-Hui Gong

**Affiliations:** 1 College of Horticulture, Northwest A&F University, Yangling 712100, Shaanxi, China; E-Mails: wjune1127@163.com (J.-E.W.); xndavid@nwsuaf.edu.cn (D.-W.L.); 2 State Key Laboratory of Crop Stress Biology in Arid Areas, Northwest A&F University, Yangling 712100, Shaanxi, China; E-Mails: hongying419@sohu.com (Y.-L.Z.); zhaoqian0225@163.com (Q.Z.); 15109240798@163.com (Y.-M.H.); 3 College of Horticulture, Henan Agricultural University, Zhengzhou 450002, Henan, China; E-Mail: kekeliu1219@sina.com

**Keywords:** pepper, peroxidase, *Phytophtora capsici*, abiotic stress, virus-induce gene silencing (VIGS)

## Abstract

Peroxidases are involved in many plant processes including plant defense responses to biotic and abiotic stresses. We isolated a novel peroxidase gene *CanPOD* from leaves of pepper cultivar A3. The full-length gene has a 1353-bp cDNA sequence and contains an open reading frame (ORF) of 975-bp, which encodes a putative polypeptide of 324 amino acids with a theoretical protein size of 34.93 kDa. *CanPOD* showed diverse expression levels in different tissues of pepper plants. To evaluate the role of *CanPOD* in plant stress responses, the expression patterns of *CanPOD* were examined using Real-Time RT-PCR. The results indicated that *CanPOD* was significantly induced by *Phytophtora capsici*. Moreover, *CanPOD* was also up-regulated in leaves after salt and drought stress treatments. In addition, *CanPOD* expression was strongly induced by signaling hormones salicylic acid (SA). In contrast, *CanPOD* was not highly expressed after treatment with cold. Meanwhile, in order to further assess the role of gene *CanPOD* in defense response to *P. capsici* attack, we performed a loss-of-function experiment using the virus-induced gene silencing (VIGS) technique in pepper plants. In comparison to the control plant, the expression levels of *CanPOD* were obviously decreased in *CanPOD*-silenced pepper plants. Furthermore, we analyzed the effect of *P. capsici* on detached-leaves and found that the *CanPOD*-silenced plant leaves were highly susceptible to *P. capsici* infection. Taken together, our results suggested that *CanPOD* is involved in defense responses to *P. capsici* infection as well as abiotic stresses in pepper plants.

## 1. Introduction

Pepper (*Capsicum annuum* L.) is an agriculturally important vegetable of global significance. It also serves as one of several model crop systems representing the family of Solanaceae. *Phytophthora capsici* is a highly dynamic and destructive pathogen of vegetables. It attacks all cucurbits, pepper, tomato and eggplant, and, more recently, snap and lima beans [1]. Plants have evolved sophisticated defense mechanisms to combat an abundance of microbial pathogens [2]. Plants have also protected themselves against severe environmental abiotic stress conditions such as cold, salt, drought and heavy metal pollutants. Environmental stresses can produce excess concentrations of reactive oxygen species, resulting in oxidative damage to, or the apoptotic death of cells [3]. In their natural environment they are often simultaneously confronted with more than one type of stress [4]. To cope with these stresses, plants have evolved to counteract the effects of ROS with antioxidants and antioxidative enzymes that maintain ROS balance within the cell. Of the antioxidative enzymes, peroxidases (POD) play key roles in cellular ROS detoxification [5].

Plant peroxidases, which are encoded by a large number of superfamily genes, are secreted from plant cells or transported into vacuoles via the endoplasmic reticulum (ER) [6,7]. In plants, peroxidases play several biological roles, including lignin biosynthesis, suberization, indole-3-acetic acid (IAA) catabolism, scavenging hydrogen peroxide or organic hydroperoxides, growth regulation and tolerance against biotic and abiotic stresses [8–14]. Due to their physiologically significant roles in plants, peroxidases have become the subject of a broad range of biochemical and molecular biological studies [15]. Peroxidases exhibit diverse expression patterns in response to environmental stresses, both abiotic and biotic, such as wounding, ethylene, pathogen infection, drought, low-temperature, iron deficiency, light and plant growth regulators, which have been well documented [9,12,14,16–23].

During the last few years, considerable progress has been made in understanding how plants protect themselves against biotic and abiotic stresses. Pathogenesis-related peroxidase gene *Shpx*2 has also been reported to enhance resistance against oomycete pathogens [24]. In *Arabidopsis thaliana*, the tissue-specific expression of 73 peroxidase genes was monitored by microarray assays [6,9]. In wheat, sequence-associated expression patterns of peroxidase genes have been demonstrated during powdery mildew (*Blumeria graminis* f.sp. *tritici*) infection [25]. Overexpression of sweet potato (*Ipomoea batatas*) *swpa*4 peroxidase significantly enhanced the salt and drought stress tolerance of tobacco plants (*Nicotiana tabacum*) [26]. More recently, overexpression of *CaPO*2 in transgenic *Arabidopsis thaliana* plants conferred enhanced tolerance to high salt, drought, and oxidative stress, while also enhancing resistance to infection by the necrotrophic fungal pathogen *Alternaria brassicicola* [27]. Do *et al.* suggested that CAPO1 may be involved in pepper defense against pathogen attack [28]. In addition, the expression of a peroxidase gene CAPO1 was highly unregulated by copper stress [29].

Although much progress has been made in elucidating the molecular and biochemical properties and physiological functions of the plant peroxidases, there is still limited knowledge because of their multitude isozyme forms, broad range of substrate specificity, complex expression profiles in response to temporal, spatial and environmental changes and the absence of simple correlations between sequence similarity and function [10]. It is necessary to improve our understanding on the molecular and functional properties of the pepper peroxidase gene *CanPOD* and to provide genetic evidence on the involvement of peroxidase in pathogen infection and abiotic stresses in pepper plants.

In this study, we isolated a peroxidase gene *CanPOD* from leaves of pepper cultivar A3. *CanPOD* showed tissue-specific expression in different organs of pepper plant. To better understand the possible roles of *CanPOD* in defense against biotic (*P. capsici* infection) and abiotic stresses (cold, salt, drought, salicylic acid and methyl jasmonate), a time course expression patterns of the *CanPOD* gene was performed using Real-Time RT-PCR. Furthermore, the loss-of-function of *CanPOD* in pepper plants was examined using a virus-induced gene silencing (VIGS) system. The results suggest that *CanPOD* may play an important role during the pathogen infection and abiotic stresses.

## 2. Results and Discussion

### 2.1. Cloning and Sequence Analysis of *CanPOD* Gene

**Figure 1:**
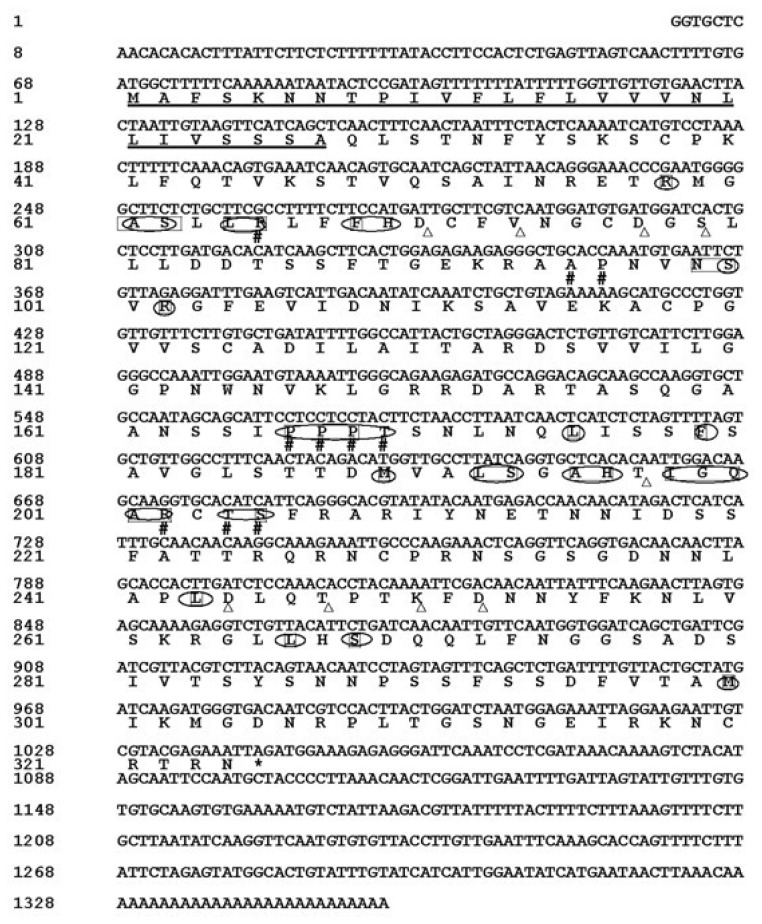
The nucleotide sequence (above) and deduced amino acid sequence (below) *CanPOD*. The nucleotide and amino acid positions are numbered on the left. The signal peptide is shown in solid line. The rectangle indicates the heme binding site; ellipse indicates the active site; # indicates the substrate binding site; Δ indicates the calcium binding sites.

To investigate the role of the peroxidase gene in pepper plant response to pathogen infection, the *CanPOD* gene was identified and characterized. The full sequence of *CanPOD* cDNA comprised of 1353-bp, containing the start code ATG, stop code TAG, a 5′-untranslated region (UTR) of 67-bp, an open reading frame (ORF) of 975-bp and a 3′-UTR of 311-bp with a poly (A^+^) tail of 28-bp. The cDNA sequence has been submitted to GenBank as accession number FJ596178. The ORF encodes a predicted protein of 324 amino acids. The calculated molecular mass of the mature protein is 34.93 kDa, with an estimated pI of 9.220. TMpred showed that *CanPOD* protein contained three inside to outside helices and one outside to inside helice, which score more than 500. Therefore, *CanPOD* protein contained transmembrane stuctures. The *CanPOD* protein would be a secretory protein with signal peptide and transmembrane structures, the resorts of NCBI Blast also sustain this viewpoint. *CanPOD* protein contained four conserved domains (Figure 1). The rectangle indicates the heme binding site with 29 residues; ellipse indicates the active site with 31 residues; # indicates the substrate binding site with 10 residues; Δ indicates the calcium binding sites with 9 residues. Eight conserved cysteine residues (C38, C71, C76, C118, C124, C203, C229 and C320) yielded four disulfide bridges.

**Figure 2:**
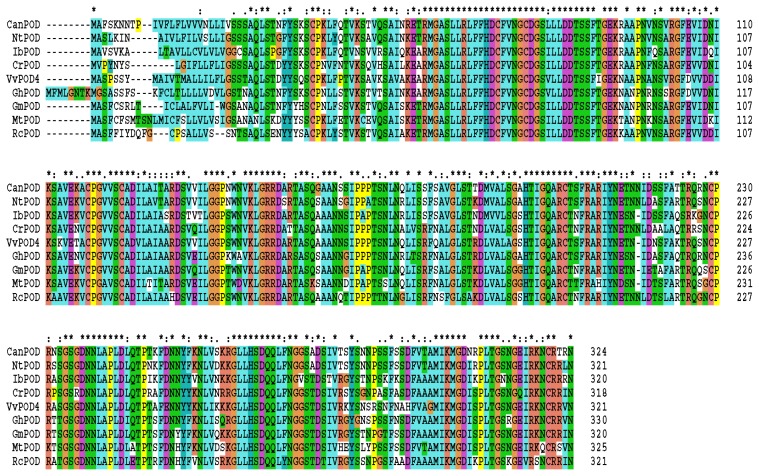
Multiple sequence alignment of amino acid sequence of *CanPOD* with amino acid sequences of *NtPOD* (*Nicotiana tabacum*, AB027752.1), *IbPOD* (*Ipomoea batatas*, AY206414.1), *CrPOD* (*Catharanthus roseus*, AY837788.2), VvPOD (*Vitis vinifera*, XM_002278960.1), *GhPOD* (*Gossypium hirsutum*, AY311597.1), *GmPOD* (*Glycine max*, XM_003523221.1), *MtPOD* (*Medicago truncatula*, XM_003616700.1) and *RcPOD* (*Rubia cordifolia*, HM807273.1). Asterisks represent identical amino acids among the different species. Period and semicolon mark positions where amino acids are similar. Gaps (-) were introduced to maximize alignment.

Multiple amino acid sequence alignment revealed that the deduced amino acid sequence of *CanPOD* displayed substantial homology with other proteins (Figure 2), including *Nicotiana tabacum* peroxidase (BAA82306.1, 88%), *Vitis vinifera* peroxidase 4 (XP_002278996.1, 78%), *Rubia cordifolia* peroxidase 6 (ADN96693.1, 78%), *Glycine max* peroxidase 52-like (XP_003523269.1, 81%), *Ipomoea batatas* anionic peroxidase swpb3 (AAP42508.1, 82%), *Gossypium hirsutum* class III peroxidase (AAP76387.1, 82%), *Catharanthus roseus* putative secretory peroxidase (AAX44001.2, 78%) and *Medicago truncatula* Peroxidase (XP_003616748.1, 77%), respectively.

**Figure 3:**
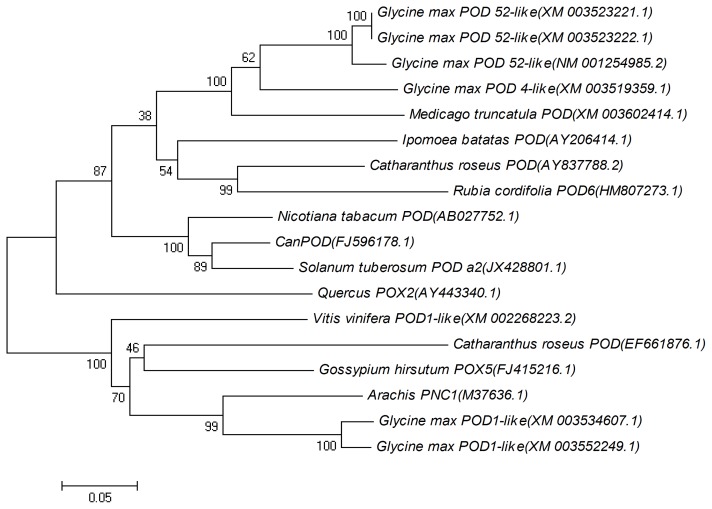
Phylogenetic tree of proteins homologous to *CanPOD* and peroxidase proteins from other species. The rooted gene tree (majority-rule consensus from 1000 bootstrap replicates) resulted from heuristic searching in Mega5.1.Bootstrap values are indicated at each branch node. GenBank accession numbers are in parentheses after each species and gene name. Scale bar indicates similarity coefficient.

To evaluate the molecular evolutionary relationships of *CanPOD* against other peroxidases, a phylogenetic tree was carried out using Mega5.1. Two clusters were formed using the full-length cDNA sequences of 18 peroxidase genes from *Catharanthus roseus*, *Arachis*, *Catharanthus roseus*, *Glycine max*, *Gossypium hirsutum*, *Ipomoea batatas*, *Medicago truncatula*, *Nicotiana tabacum*, *Quercus*, *Rubia cordifolia*, *Solanum tuberosum* and *Vitis vinifera* (Figure 3). *CanPOD* gene (FJ596178) was grouped together with POD a2 from *Solanum tuberosum*, which is a secretory peroxidase, belongs to the plant peroxidase super family. All of the bioinformatics analysis results suggested that *CanPOD* should be a plant peroxidase.

### 2.2. Tissue-Specific Expression of *CanPOD* Gene in Different Tissues of Pepper Plants

**Figure 4:**
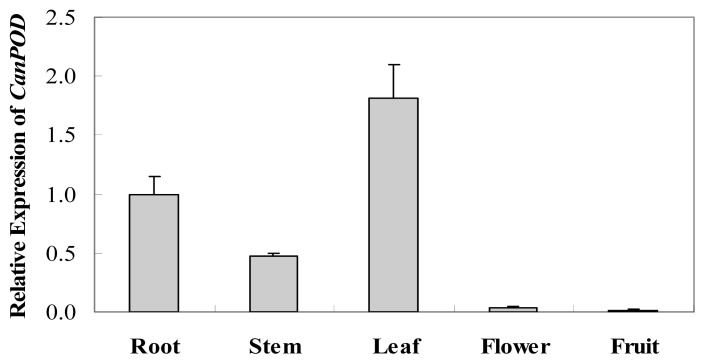
Tissue-specific expression of *CanPOD* gene in different tissues of pepper plants. Error bars represent the mean ± SD of three independent biological replicates.

In order to investigate the expression levels of *CanPOD* gene in different tissues of pepper cultivar A3 plants, total RNA was extracted from roots, stems, leaves, flowers and immature fruits. The results were shown in Figure 4. Differences were observed among the different tissues of pepper plants. In general, the highest expression levels of *CanPOD* gene were detected in leaves, followed by roots, stems and flowers, respectively. However, *CanPOD* gene was slightly expressed in immature fruits. The results suggest that *CanPOD* gene is differentially expressed in the tissues of pepper cultivar A3 plant. This finding is in agreement with previous studies showing that the expression of many peroxidase genes is tissue specific [6,7,9,14,16,20]. The relatively high expression of *CanPOD* gene in leaves suggests that *CanPOD* gene may be associated with defense responses in leaves.

### 2.3. Expression Profiles of *CanPOD* Gene in Response to Biotic and Different Aboitic Stress Treatments

Plants possess an elaborate and finely regulated defense system to defend against various biotic and abiotic stresses [27]. To study the relationship between the *CanPOD* gene and stress response of pepper, the expression patterns of *CanPOD* gene in response to diverse stresses were measured, including biotic stress (pathogen infection) and abiotic stresses (cold, salt, drought, SA and MeJA), using Real-Time RT-PCR.

#### 2.3.1. Expression Analysis of *CanPOD* Gene in Response to *Phytophthora capsici* Infection

**Figure 5:**
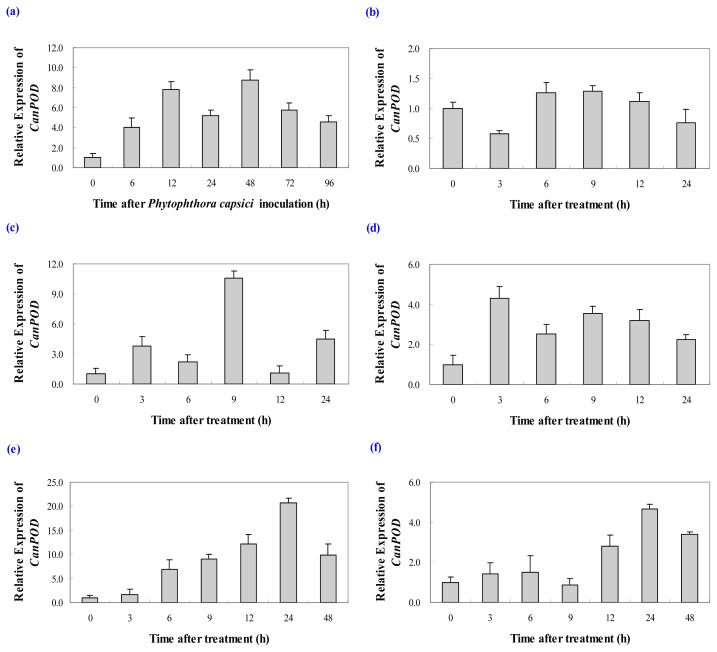
Real-Time RT-PCR analysis of relative *CanPOD* gene expression levels in leaves of pepper plants after biotic and different abiotic stress treatments. (**a**) *Phytophthora capsici* Infection; (**b**) Cold stress; (**c**) Salt stress; (**d**) Drought stress; (**e**) SA stress and (**f**) MeJA stress. Error bars represent the mean ± SD of three independent biological replicates.

It have been reported that the peroxidases are known to be induced by various pathogens infection [3,14,27]. To explore whether *CanPOD* is involved in pathogen defense, the expression levels of this gene were measured in leaves after inoculation with the avirulent strain of *P. capsici*. The results from Real-Time RT-PCR showed that the *CanPOD* gene transcripts were strongly induced in the incompatible interaction with *P. capsici* (Figure 5a). Increased levels of *CanPOD* transcript was detectable at 6 h after inoculation, and maintained for 12 h and reached the maximal level at 48 h after inoculation. From 48 to 96 h, the expression levels of *CanPOD* gene presented a decreased trend. However, compared with the control plants, the expression levels of *CanPOD* gene were up-regulated in leaves in response to inoculation with the avirulent strain of *P. capsici*. Infection of rice leaves by *Xanthomonas oryzae* pv*. oryzae*, the causal pathogen of rice blight, strongly induces a Peroxidase isoform in xylem vessels, which results in secondary wall thickening and reduced access of the pathogen to the pit membrane, which is the pathogen’s contact point in living cells [8]. Do *et al.* reported that the strong accumulation of *CAPOA*1, *CAPOT*1 and *CAPO*1 transcripts in the incompatible interaction was induced in the pepper leaves infected by *P. capsici*, which suggests that the peroxidase gene expression may be related to ROS-associated defense response of pepper to *P. capsici* infection [28]. Sarowar *et al.* showed that *CAPOA*1-overexpressed tobacco plants exhibited increased resistance to the oomycete pathogen, *Phytophthora nicotianae* [3]. However, the transgenic plants were not found to be resistant to the bacterial pathogen, *Pseudomonas syringae* pv. *tabaci*, but were weakly resistant against the bacterial pathogen *R. solanacearum*. A previous study demonstrated that overexpression of *CaPO*2 in *Arabidopsis* were to exhibit enhanced resistance to *P. syringae* pv. *tomato* DC3000 infection [14]. Choi *et al.* reported that the *CaPO*2-OX lines exhibited enhanced resistance to infection by the necrotrophic fungal pathogen *A. brassicicola*, but not to *H. arabidopsidis* isolate Noco2 [27]. This suggests that, although some peroxidases are clearly involved in host resistance to certain pathogen, not all the peroxidases play such a defensive role. When considering these previous studies, our results indicate that expression of *CanPOD* is very rapidly induced upon pathogen infection, and suggesting that *CanPOD* might be play an important role in the pepper plant defense response to *P. capsici* attack.

#### 2.3.2. Expression Analysis of *CanPOD* Gene in Response to Cold, Salt and Drought Stress Treatments

Plant peroxidases are known to function as an important antioxidant system, along with superoxide dismutase, catalase and glutathione reductase. They play a role in combating the oxidative stress induced by cold, salt and drought [5,30,31]. To evaluate the possible roles of the *CanPOD* in the defense response of pepper against various abiotic (cold, salt and drought) stresses, the *CanPOD* expression analysis was carried out by Real-Time RT-PCR using gene-specific primers. The results showed diverse expression patterns in response to cold, salt and drought stresses (Figure 5b–d).

Temperature stress raises ROS levels in plants and would be expected induce various kinds of antioxidant enzymes to overcome the resulting oxidative stress [32]. In our study, the *CanPOD* transcript levels were somewhat increased in response to cold stress. During the first 3 h, the expression of *CanPOD* decreased down to 42% of the control. As time progressed, the expression level of *CanPOD* was gradually increased and reached a peak (1.7-fold) at 9 h. After that, the *CanPOD* expression level began to decrease. At 24 h, *CanPOD* mRNA level was only 0.7-fold compared with the control. On the contrary, Choi found that cold stress strongly induced the *CaPO*2 gene in pepper leaves up to 25 h after treatment [27]. Park reported that six POD genes showed different expression levels in response to chilling, which only gene *swpa*4 was strongly expressed at 4 °C. However, the expression level of *swpb*3 was decreased [20]. These results suggest that there exist changes in gene expression patterns under cold stress.

In contrast to the results for cold stress, the *CanPOD* gene expression levels were significantly enhanced in pepper leaves under salt stress, showing a 10.5-fold induction at 9 h after 0.4 M NaCl treatment. Moreover, the *CanPOD* was up-regulated throughout the entire treatment period (Figure 5c). Consistent with these results, previous studies also showed that *POD* genes are up-regulated by salinity stress in plants [27,33,34]. Therefore, we conclude that the *CanPOD* gene involves in pepper plant resistance to salt stress.

Previous studies demonstrated upregulation of *POD* genes in response to drought stress, and transgenic plants expressing exogenous *POD* genes showed high levels of tolerance to drought stress [26,35]. In our study, the expression levels of *CanPOD* were also up-regulation in response to drought stress. However, the *CanPOD* expression levels was substantially lower than the one in response to salt stress, and reached maximum 4.3-fold at 3 h (Figure 5d). The result is in accordance with the results of Choi that the *CaPO*2 gene was strongly induced by drought stress [27]. However, other researched proposed the different outcome that *PoPOD*1 expression was down-regulated by PEG, which induce the generation of ROS in plant cells [15,36,37]. These results suggest that the pepper peroxidase gene *CanPOD* may be inducible during the drought stress.

#### 2.3.3. Expression Analysis of *CanPOD* Gene in Response to SA and MeJA Treatments

Various plant hormones, such as salicylic acid (SA) and methyl jasmonic acid (MeJA), are important signaling molecules that contribute to the establishment of disease resistance and abiotic stress tolerance in pepper plants [38–40]. SA typically mediates basal defence to biotrophic pathogens, while JA generally controls defense reactions to necrotrophs [41]. To determine whether these defense-related signaling molecules can induce *CanPOD* gene expression, we performed Real-Time RT-PCR in pepper leaves treated with 5 mM SA or 50 μM MeJA.

Response to SA stress treatment, the expression of *CanPOD* was induced dramatically (Figure 5e). The *CanPOD* transcripts accumulated as early as 3 h after treatment, and presented a gradual increase. The highest transcript levels of *CanPOD* occurred (20.7-fold) at 24 h, comparing to the control. There was a sharply decrease at 48 h, which was the last observed time point (Figure 5e), indicated that *CanPOD* expression was considerably induced or up-regulated by the signal molecule SA. In contrast, Choi *et al.* reported that SA cannot induce *CanPOD* gene expression [27]. SA (20 μM), which controls the systemic acquired plant pathogen response, did not affect the steady-state levels of *PoPOD*1 transcripts [15]. These opposite opinions may be due to SA have been shown to activate different sets of plant PR genes and act either synergistically or antagonistically during defense signaling dependent on their concentrations [42].

To assess the possible involvement of *CanPOD* in signaling pathways utilized by MeJA stress, 50 μM MeJA was sprayed as the same method used for the SA stress. As shown in Figure 5f, *CanPOD* transcript levels were slowly increased between 3 and 6 h after treatment, but they decreased to the basal level at 9 h. The strongest response to MeJA was observed (4.6-fold) at 24 h, compared to the control. Similarly, MeJA enhanced the expression of some POD genes from sweetpotato, such as *swpa*4, *swpa*5 and *swpb*2 [29]. However, there was a different result that expression of the wound-inducible basic *POD* gene *tpoxN*1 was not enhanced by MeJA in tobacco plants [16]. These results suggest that while *CanPOD* gene may be involved in the JA-dependent signaling pathway.

Taken together with these results, we demonstrated that *CanPOD* may be potentially valuable gene for the genetic engineering of plants for pathogen infection as well as abiotic stress resistance.

### 2.4. Virus-Induced Gene Silencing (VIGS) of *CanPOD* Gene in Pepper Plants

**Figure 6:**
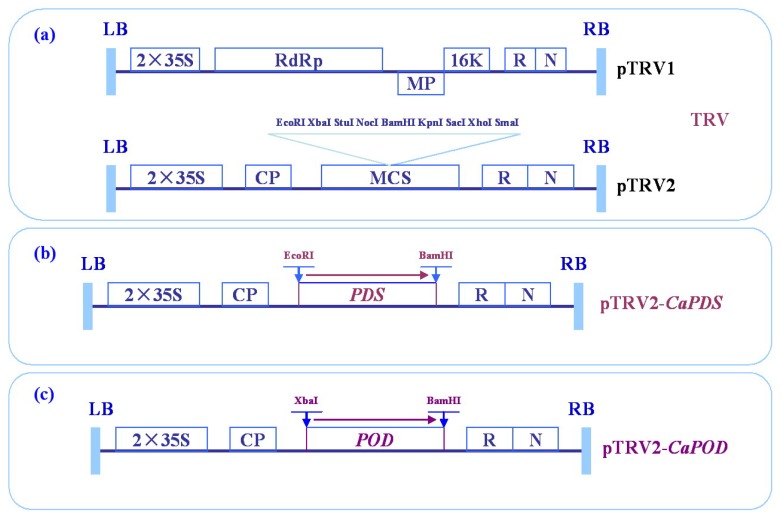
Schematic representation of the tobacco rattle virus (TRV), pTRV2-*CaPDS* and pTRV2-*CanPOD* constructs. LB: left borders of the T-DNA; RB: right borders of the T-DNA; 2 × 35S: two copies of the cauliflower mosaic virus 35S promoter; CP: coat protein; RdRp: RNA-dependent RNA polymerase; MP: movement protein; 16K: 16 KDa protein; R: ribozyme; N: nos-terminator; MCS: multiple cloning sites. (**a**) TRV vector; (**b**) pTRV2-*CaPDS* vector; (**c**) pTRV2-*CanPOD* vector.

To examine the effect of loss-of-function of the *CanPOD* gene in pepper plants, VIGS was performed in pepper cultivar A3 using the tobacco rattle virus (TRV)-based virus-induced gene silencing (VIGS) technique [14,43–46]. A fragment from the 3′ end of the *CanPOD* open reading frame was cloned into the pTRV2 vector and generated pTRV2-*CanPOD* vector (Figure 6). Empty vector (pTRV-00) was used as a negative control.

**Figure 7:**
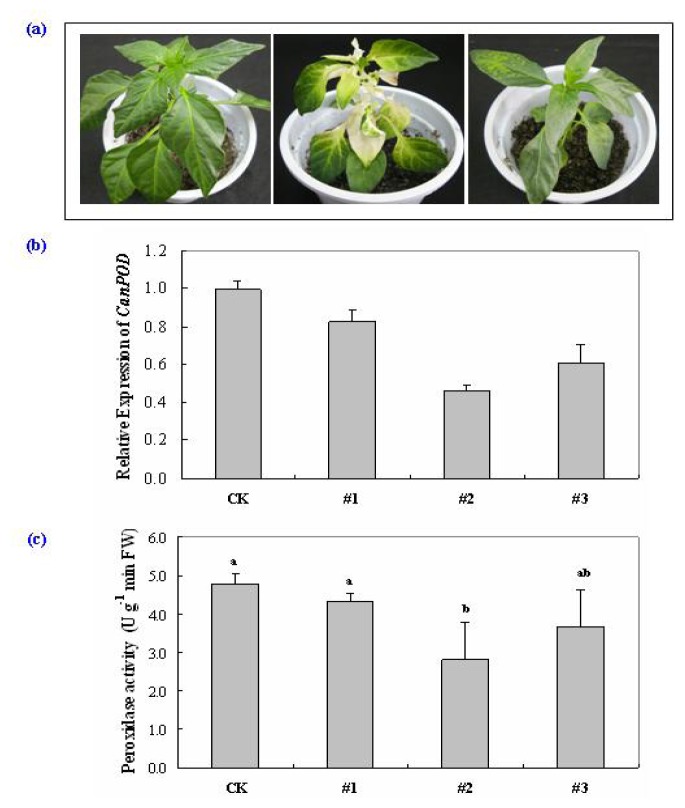
Eeffect of *CanPOD* gene silencing on pepper plants. The photographs were taken 35 d after inoculation. (**a**) Phenotypes of gene silencing pepper plants. Left: control plant (pTRV2-00); Middle: *CaPDS*-silenced plant (pTRV2-*CaPDS*); Right: *CanPOD-*silenced plant (pTRV2-*CanPOD*). (**b**) Real-Time RT-PCR analysis of *CanPOD* expression levels in control (pTRV2-00) and silenced (pTRV2-*CanPOD*) pepper plants (#1, #2 and #3) 35 d after inoculation. (**c**) Peroxidase (POD) activities in leaves of the control plant (pTRV2-00) and the silenced (pTRV2-*CanPOD*) pepper plants (#1, #2 and #3) 35 d after inoculation. Error bars represent the mean ± SD of three independent biological replicates. Letters indicate significant differences using Fisher’s LSD test at *p* ≤ 0.05.

The pTRV2-*CaPDS*, which silences phytoene desaturase gene (*PDS*) and induces a photo-bleaching phenotype, was used as a positive control to determine the success of gene silencing. As shown in Figure 7a, symptoms of photo-bleaching were occurred on the leaves of *CaPDS* silenced plants. This provided the evidence that VIGS is successful in our study. There was no morphological difference between the control plant (pTRV-00) and the *CanPOD* silenced plant (pTRV2-*CanPOD*) 35 d after inoculation (Figure 7a). Meanwhile, the efficiency of the VIGS was investigated by Real-Time RT-PCR (Figure 7b). The results revealed that the levels of *CanPOD* expression in *CanPOD* silenced plant (pTRV2-*CanPOD*) were reduced drastically comparing to the control pepper plant (pTRV-00). These RT-PCR data indicate that *CanPOD* was silenced effectively in pepper plants. In order to further confirm the efficiency of VIGS on *CanPOD*, the peroxidase (POD) enzymatic activity in pepper leaves was tested in the control plant (pTRV-00) and *CanPOD* silenced plant (pTRV2-*CanPOD*). As shown in Figure 7c, the activities of POD enzyme were decreased in the *CanPOD* silenced plants comparing to the control plant, which were essentially identical to the results from the Real-Time RT-PCR, proving the validity of RT-PCR analysis. These findings further suggest that VIGS was successful and effective for *CanPOD* silencing on pepper plants.

**Figure 8:**
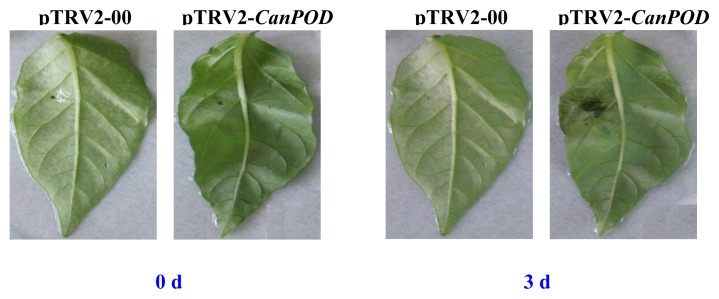
Disease symptoms in detached-leaves of the control plant (pTRV2-00) and the silenced pepper plant (pTRV2-*CanPOD*) after inoculation with *P. capsici*.

It is well known that peroxidase be induced by various pathogens infection [12,14,20,27]. The *CanPOD* expression in pepper leaves was significantly induced in the incompatible interaction with avirulent strain of *P. capsici*. To determine the role played by *CanPOD* in the basal defense response of pepper, we analyzed the levels of resistance to *P. capsici* infection in leaves of *CanPOD* silenced plants. Five weeks after VIGS, the fourth leaves from the top of control plants (pTRV2-00) and silenced pepper plants (pTRV2-*CanPOD*) were detached and inoculated by addition of 20ul zoospores suspension (10^4^ cfu mL^−1^) of *P. capsici* by using a syringe without needle. Disease symptoms were observed in the *CanPOD* silenced plant leaves 3 d after inoculation. However, the control (pTRV-00) plant leaves did not exhibit any cell death or disease symptoms (Figure 8). Therefore, we demonstrate that *CanPOD*-silenced plants are more susceptible to *P. capsici* infection than the control plants. One paper had reported similar results to our study [14]. As a whole, the *CanPOD* gene is involved in the defense responses to pathogen infection of pepper plants.

## 3. Experimental Section

### 3.1. Plant Materials and Growth Conditions

Pepper cultivar A3 was used in this study, which provided by pepper breeding group in Northwest A&F University, China. Seeds were soaked in warm water (55 °C) for approximately 20 min to promote germination. The seeds were rinsed twice every day and then placed on moist gauze in an incubator (28 °C, 60% relative humidity in darkness). When the seeds were at least 80% germinated, they were sown in a soil mix [grass charcoal/perlite (3/1, *v*/*v*)] in plastic pots. Pepper plants were grown in a growth chamber with a 16 h light and 8 h dark photoperiod at 25 °C.

### 3.2. Pathogen Preparation and Inoculation Procedures

Preparation of *P. capsici* inocula was described previously [47]. The avirulent strain PC of *P. capsici* was grown on potato dextrose agar (PDA) medium in darkness at 28 °C for 7 days. The mycelia were scraped and incubated under fluorescent light for 2 days to promote sporangium formation. Zoospores were prepared by sporulating cultures with sterile distilled water and incubating at 4 °C for 1 h followed by 30 min at 28 °C to initiate zoospore release. The zoospores were collected by filtering through four layers of cheesecloth and estimated using a hemacytometer. The concentration of the zoospore suspension was adjusted to 1 × 10^4^ zoospores per millimeter with sterile water.

For pathogen stress treatment, pepper plants at six-true leaf stage were inoculated by addition of 2.5 mL zoospores suspension (10^4^ cfu. mL^−1^) of *P. capsici* each pot.

For virus-induced gene silencing (VIGS) of *CanPOD* gene in pepper plants, the fourth leaves from the top of control plants (pTRV2-00) and silenced pepper plants (pTRV2-*CanPOD*) after inoculation 5 weeks were collected and sterilized with 75% ethanol for 1 min, washed three times with sterile water, then injected by addition of 20ul zoospores suspension (10^4^ cfu. mL^−1^) of *P. capsici* by using a syringe without needle. After inoculation, the leaves were grown in a growth chamber at 28 °C under a 16 h light/8 h dark photoperiod cycle with 60% relative humidity.

### 3.3. Cloning and Sequence Analysis of *CanPOD* Gene

Total RNA was extracted from young leaves of pepper cultivar A3 using the Trizol (Invitrogen) method. To isolate the complete 5′ and 3′ regions of the putative *CanPOD* gene, the rapid amplification of cDNA ends (RACE) method was used. First-strand cDNA synthesis was performed using Smart RACE cDNA amplification kit (Clontech). Gene-specific primer GSP1: (5′-GCTTCGTCAATGGATGTGATGGA-3′) was used for 3′-RACE and the gene-specific primer GSP2: (5′-TGGAAGAAAAGGCGAAGCAGAGA-3′) was used for 5′-RACE, respectively. The universal primers for 5′ and 3′ RACE are given in the kit. The full-length cDNA sequence of *CanPOD* gene was obtained by PCR amplification using forward (5′-GGTGCTCAACACACACTTTA-3′) and reverse (5′-TTGTTTAAGTTATTCATGAT-3′) primers. The PCR products were cloned into pMD19-T vector (Takara) and sequenced by Shanghai GeneCore Biotechnologies Co. (GeneCore).

NCBI bioinformatics tools [48] were used to compare and analyze the nucleotide and protein sequences data. Multiple sequence alignments of *CanPOD* gene with peroxidase from other species were performed by the Clustal X1.83 analysis program of DNAstar software (LaserGene, Madison, WI, USA). The phylogenetic tree was constructed using Mega5.1 by the neighbor-joining method. Conserved domains in protein were identified in Conserved Domain database [49].

### 3.4. *CanPOD* Gene Expression Patterns Analysis

#### 3.4.1. Tissue-Specific Expression of *CanPOD Gene*

To evaluate the expression levels of *CanPOD* gene in different organs of pepper plants under normal conditions, root, stem, leaf, flower, and immature fruit samples were collected from pepper cultivar A3 plants, then frozen in liquid nitrogen and kept at −80 °C for further gene expression analysis.

#### 3.4.2. Stress Treatments

Pepper plants at the six-true leaf stage were used for biotic stress and treatments with environmental stresses and plant hormone.

For pathogen treatment, pepper plants were inoculated by addition of 2.5 mL zoospores suspension (10^4^ cfu. mL^−1^) of the avirulent strain of *P. capsici*. Mock plants were inoculated with distilled water. Leaves from non-infected and infected plants were collected at 0, 6, 12, 24, 48, 72 and 96 h after inoculation, immediately frozen in liquid nitrogen and kept at −80 °C.

For cold stress treatment, pepper plants were placed at 4 °C for low temperature treatment. For salt and drought stress treatments, the seedlings were watered into their roots with solutions of 0.4 M sodium chloride (NaCl) or 0.4 M mannitol. Control plants were treated with sterile ddH_2_O. Leaves from mock and stress-treated plants were collected at 0, 3, 6, 9, 12 and 24 h after treatment, immediately frozen in liquid nitrogen and kept at −80 °C.

For plant hormone treatments, pepper plants were sprayed with 5 mM salicylic acid (SA) or 50 μM methyl jasmonate (MeJA) solutions. Mock plants were sprayed with sterile ddH_2_O and placed in a separate chamber. Leaves from mock and treated plants were collected at 0, 3, 6, 9, 12, 24 and 48 h after treatment, immediately frozen in liquid nitrogen and kept at −80 °C.

All treatments were placed in a growth chamber at 25 °C (except cold stress) under a 16 h light/8 h dark photoperiod cycle with 60% relative humidity. All treatments were performed and analyzed triplicate in separate experiments.

### 3.5. RNA Isolation and Real-Time RT-PCR Analysis

Total RNA was extracted from pepper leaves of different time points after diverse stress treatments using Trizol (Invitrogen) method. The concentration of total RNA was measured spectrophotometrically using a NanoDrop instrument (Thermo Scientific NanoDrop 2000C Technologies, Wilmington, DE, USA), and the purity was assessed using the A260/280 and A260/230 ratios provided by NanoDrop. For quantitative Real-Time RT-PCR analysis, first strand cDNA was synthesized from 500 ng total RNA using a PrimeScript™ Kit (TaKaRa, Bio Inc, China) following the manufacturer’s protocols. Real-Time RT-PCR was carried out using SYBR® Premix Ex Taq™ II (TaKaRa, Bio Inc., China). Real-Time RT-PCR analysis was performed on a 20 μL mixture containing 10.0 μL SYBR^®^ Premix Ex Taq™ II, 2.0 μL diluted cDNA, 0.8 μL of forward and reverse primers. The amplification was completed with the following cycling parameters: 95 °C for 1 min, followed by 45 cycles at 95 °C for 10 s, 52 °C for 30 s, and 72 °C for 30 s. The gene of *Ubi3* (AY486137.1) was used as internal control (reference gene) in this study [50]. The primer sequences used for Real-Time RT-PCR were shown in Table 1. The relative expression levels of gene were determined by using comparative threshold method of 2^−ΔΔCt^ [51]. All samples were performed in triplicate and each had at least three independent biological replicates.

### 3.6. Virus-Induced Gene Silencing (VIGS) Analysis of *CanPOD* Gene in Pepper Plants

The tobacco rattle virus (TRV)-based VIGS system was used for gene silencing in pepper plants as described by [14,52]. Phytoene desaturase (*PDS*) encodes an enzyme involved in the carotenoid biosynthesis pathway [53]. The *CaPD*S (*phytoene desaturase* from *C. annuum*, GenBank: X68058.1) has been used as a visual marker for the effectiveness of VIGS in pepper plants. A fragment of coding region of *CaPDS* gene was amplified using gene-specific primers (Forward: 5′-GGGGAATTCTGTTGTCAAAACTCCAAGGTCTGTA-3′ with an *Eco*RI restriction site and Reverse: 5′-GGGGGATCCTTTCTCCCACTTGGTTCACTCTTGT-3′ with a *Bam*HI restriction site). The resulting product was inserted into pTRV2 vector to generate pTRV2-*CaPDS*. A fragment of coding region of *CanPOD* gene was amplified using gene-specific primers (Forward: 5′-GGGTCTAGAGTGCTCAACACACACTTTATTCTTCTC-3′ with an *Xba*I restriction site and Reverse: 5′-GGGGGATCCCCAAGAATGACAACAGAGTCCCTA-3′ with a *Bam* HI restriction site). The resulting product was inserted into pTRV2 vector to generate pTRV2-*CanPOD*. The pTRV1, pTRV2, pTRV2-*CaPDS* and pTRV2-*CanPOD* vectors were transformed into the *Agrobacterium tumefaciens* strain GV3101, respectively. *Agrobacterium tumefaciens* strain GV3101 carrying pTRV1 were respectively mixed with pTRV2, pTRV2-*CaPDS* and pTRV2-*CanPOD* at a 1:1 ratio. The suspensions of *agrobacterium* inocula containing pTR1 and pTRV2-00, pTRV2-*CaPDS* or pTRV2-*CanPOD* (OD_600_ = 1.0) was infiltrated into the fully expanded cotyledons of pepper plants using a 1.0 mL sterilized syringe without a needle. The *agrobacterium-*inoculated pepper plants were grown in a growth chamber at 18 °C in darkness for 2 days with 45% relative humidity, and then transferred into a growth chamber at 22 °C under a 16 h light/8 h dark photoperiod cycle with 60% relative humidity. Leaves of control plants (pTRV2-00) and silenced pepper plants (pTRV2-*CanPOD*) after inoculation five weeks were used for *CanPOD* gene analysis.

### 3.7. Peroxidase (EC 1.11.1.7) Activity Assay

Activities of Peroxidase (POD) enzyme were quantified using the technique of Beffa [54]. The samples were collected from leaves of control plants (pTRV2-00) and silenced plants (pTRV2-*CanPOD*) 35 d after inoculation and stored at −80 °C. The lyophilised leaf powder (approximately 1.0 g) was ground in a mortar and homogenised with 5.0 mL pre-chilled extraction buffer (0.1 M potassium phosphate buffer (pH 7.5), 1mM EDTA, and 4% polyvinylpyrrolidone). The homogenate was centrifuged at 12,000 rpm for 25 min. The supernatant fraction was used as a crude extract for the enzyme activity assays. All procedures were carried out at 4 °C. The reaction mixtures contained 25 μL 50 mM H_2_O_2_, 5 μL 250 mM guaiacol, 195 μL 12.5 mM 3,3-dimethylglutaric acid (3,3-DGA)-NaOH (pH 6.0) and 25 μL enzyme extract. The optical density was recorded at 470 nm. The amount of enzyme required for the formation of 1 μmol tetraguaiacol min^−1^ at room temperature was defined as 1 unit (U) of PX activity. Each treatment was conducted in three independent experiments and each measurement was repeated three times.

### 3.8. Statistical Analysis

All data were expressed as the mean ± SD of three independent replicates (*n* = 3). Data from replicates of the same experiment were pulled together for one-way analysis of Variance (ANOVA), and differences among means of treatments were determined using the least significant difference (LSD). Statistical procedures were performed using Statistical Analysis System software (SAS Institute, version 8.2). Values of *p* ≤ 0.05 were considered statistically significant.

## 4. Conclusions

In conclusion, we isolated a peroxidase gene, named *CanPOD*, from leaves of pepper cultivar A3. *CanPOD* transcript level is diverse in different organs. The expression patterns of *CanPOD* were examined in leaves of pepper plants during biotic and abiotic stresses. The results indicated that *CanPOD* was strongly induced upon inoculation with *P. capsici*. Moreover, *CanPOD* expression was significantly up-regulated by SA treatment. Thus, expression analyses demonstrated that the *CanPOD* gene was also involved in other abiotic stresses, including salt, drought and MeJA. However, the *CanPOD* gene was not evidently expressed under cold stress comparing to the other abiotic stresses. In addition, virus-induced gene silencing (VIGS) of *CanPOD* revealed that it was more susceptible to *P. capsici* attack in *CanPOD*-silenced leaves than in the control plants. On the basis of these studies, we will perform genetic transformation to further explore the functional role of *CanPOD* in disease resistance of pepper plants. Taken together, these results suggest that *CanPOD* playes an important role in defense response to *P. capsici* infection and abiotic stresses.

## Figures and Tables

**Table 1:** Primer sequences used for Real-Time RT-PCR analysis.

Gene	Primer	Sequence (5′-3′)
*CaUbi*3	Q*CaUbi*3-F	TGTCCATCTGCTCTCTGTTG
	Q*CaUbi*3-R	CACCCCAAGCACAATAAGAC
*CanPOD*	Q*CanPOD*-F	CAAGGTTCAATGTGTGTTACC
	Q*CanPOD*-R	ATGATGATACAAATACAGTGCC

F: forward primer; R: reverse primer.
